# Systematic proteomics profiling of lysine crotonylation of the lung at Pseudoglandular and Canalicular phases in human fetus

**DOI:** 10.1186/s12953-023-00215-8

**Published:** 2023-12-01

**Authors:** Wei Wang, Wei Shi, Yinglan Wang, Yane Yang, Ping Li, Zhipeng Zeng, Wenlong Hu, Yumei Chen, Donge Tang, Yong Dai

**Affiliations:** 1 Clinical Medical Research Center, Guangdong Provincial Engineering Research Center of Autoimmune Disease Precision Medicine, Shenzhen Engineering Research Center of Autoimmune Disease, The Second Clinical Medical College of Jinan University, The First Affiliated Hospital Southern University of Science and Technology, ShenzhenPeople’s Hospital, Shenzhen, Guangdong 518020 People’s Republic of China; 2 Department of Obstetrics and Gynecology, The Second Clinical Medical College of Jinan University, The First Affiliated Hospital Southern, University of Science and Technology, ShenzhenPeople’s Hospital, Shenzhen, Guangdong 518020 People’s Republic of China; 3 Shenzhen Far East Women & Children Hospital, Shenzhen, 518000 Guangdong China

**Keywords:** Lung development, Proteomics, Lysine crotonylation, Epigenetics, Bioinformatics

## Abstract

Lung tissue is an important organ of the fetus, and genomic research on its development has improved our understanding of the biology of this tissue. However, the proteomic research of developing fetal lung tissue is still very scarce. We conducted comprehensive analysis of two developmental stages of fetal lung tissue of proteomics. It showed the developmental characteristics of lung tissue, such as the down-regulation of metabolism-related protein expression, the up-regulation of cell cycle-related proteins, and the regulation in proteins and pathways related to lung development. In addition, we also discovered some key core proteins related to lung development, and provided some key crotonylation modification sites that regulation during lung tissue development. Our comprehensive analysis of lung proteomics can provide a more comprehensive understanding of the developmental status of lung tissue, and provide a certain reference for future research and epigenetics of lung tissue.

**Supplementary Information:**

The online version contains supplementary material available at 10.1186/s12953-023-00215-8.

## Introduction

Fetal development is crucial to the survival of newborns, among which lung hypoplasia is one of the main findings of autopsy of children who have died during the neonatal period [1]. Lung development had divided into embryonic stage, fetal stage, and postpartum lung development. The fetal stage includes Pseudoglandular, Canalicular, Saccular, and Alveolarization. The stages of lung development are mainly based on morphological criteria [2]. Nevertheless, lung development is very complex, and all stages overlap. In addition, cell metabolism, growth, and differentiation are involved in the process of lung development. However, research on fetal lung development is still limited.

Crotonylation is a new type of acylation that first discovered by the research team of Professor Yingming Zhao of the University of Chicago in 2011, and selected as the annual research highlight in the field of epigenetics by Cell magazine. It refers to the introduction of crotonyl groups on the amino acid residues of histones, mainly in the lysine residues of histones, and participates in important biological processes such as gene expression regulation. Crotonylation is evolutionarily conserved, usually combined with transcriptionally active chromatin regions, and plays an important role in regulating gene expression. Crotonylation closely related to reproductive regulation, and had shown to maintain the activity of sex chromosome-related genes in meiotic metaphase.

Now, proteomics technology has been widely used in the research and exploration of biological sciences [3]. Although many analyses describing gene expression in normal lung development have been reported [4, 5], there are few protein aspects. Meanwhile, the research on lung development has used mouse models for proteomic analysis, which provides guidance for human lung development to a certain extent [6], there is no report on human fetal lung development. Secondly, protein post-translational modification (PTM) is a vital regulation of protein function, which is essential to the structure and function of protein under physiological and pathological conditions, such as gene expression and regulation, cell growth, embryonic development, metabolism, and disease treatment [7]. Among them, the crotonylation modification is genetically related, and it had reported to have important significance in cell differentiation and metabolism [8, 9]. However, it had not reported in lung development.

In this study, we combined proteomics and crotonylation modification omics to analyze the two stages of lung development (Pseudoglandular and Canalicular), revealing regulation in proteins and modifications during lung development, especially metabolism, Cell cycle, and differentiation. We provided comprehensive insights into the biological functions of proteins and crotonylation modifications in lung development, which may pave the way for the identification of the stages of lung development and the mechanisms of lung hypoplasia.

## Results

### Protein difference between Pseudoglandular and Canalicular in the human fetal lung

**Fig. 1:**
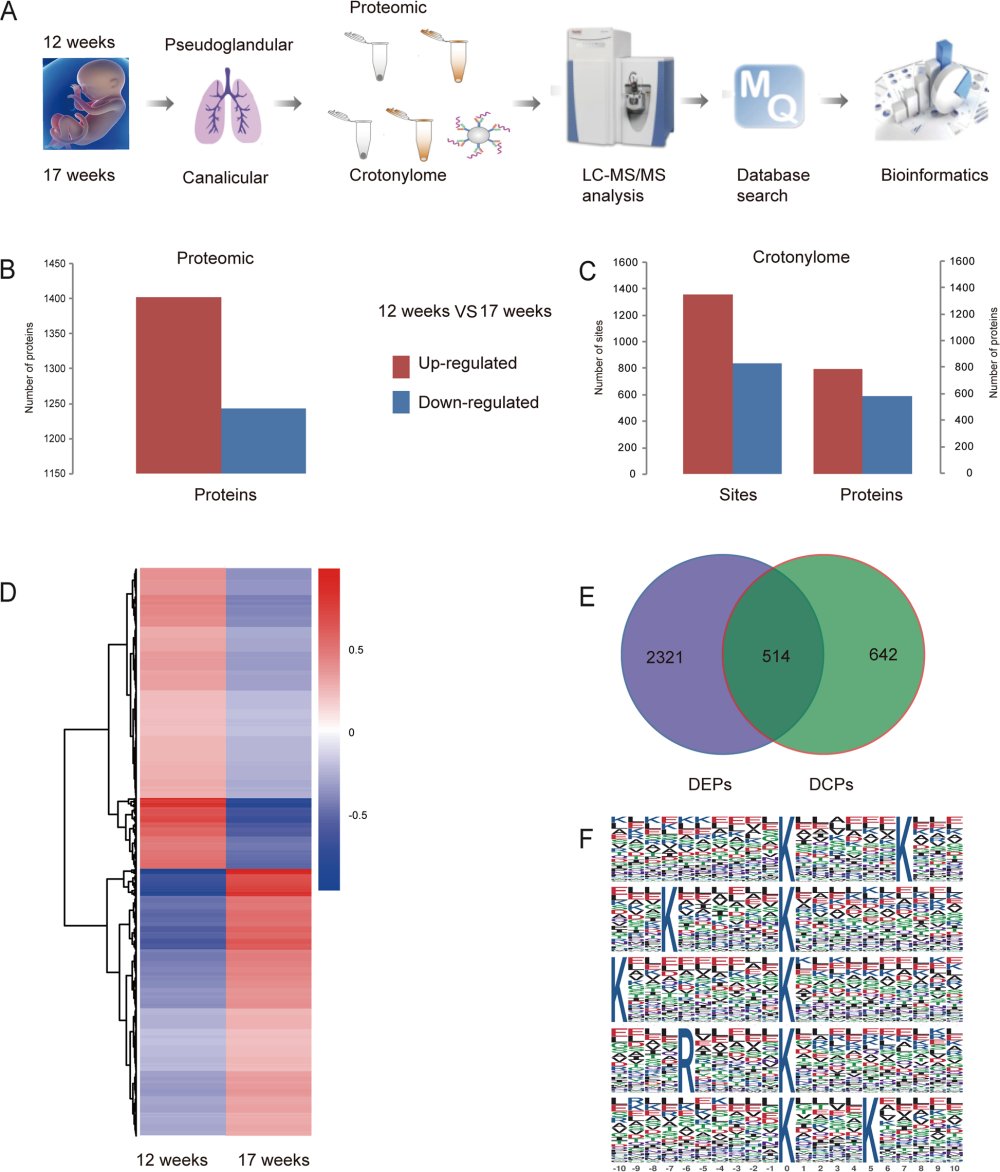
Numerous Proteins Differentially Expressed in the Lung of Fetal Were Identified. **A** The schematic flow to study the proteome and crotonylome of fetal lung. **B**, **C** DEPs and DCPs between 12 and 17 weeks of fetal lung, respectively. **D** Hierarchical clustering analysis of DEPs. **E** Venn diagram between DEPs and DCPs. **F** Significantly enriched crotonylation motifs of top 5

An overview of the experimental measure had shown in Fig. 1A. We used Label Free technology to identified 6,943 proteins (5,929 quantitative proteins) for protein expression to Pseudoglandular and Canalicular in LC–MS/MS. Compared with the fetal lung of 17 weeks, there were 2,645 DEPs (different expression proteins) (Fig. 1B), including 1,402 proteins up-regulated (≥ 1.5-fold) and 1243 down-regulated (≤ 0.67-fold) in the fetal lung of 12 weeks. For protein crotonylation, we identified 15,427 crotonylation sites in 3,802 proteins (6,960 quantitative crotonylation sites in 2,217 proteins). Among then, 2,196 crotonylation sites were identified in 1,156 DCPs(different crotonylation proteins) between the fetal lung of 17 weeks and 12 weeks (Fig. 1C). One thousand three hundred fifty-nine crotonylation sites were up-regulated (≥ 1.5-fold) in 793 proteins, and 837 crotonylation sites were down-regulated (≤ 0.67-fold) in 586 proteins. The hierarchical clustering analysis of DEPs had shown in Fig. 1D. We overlapped to 514 proteins in all DEPs and DCPs (Fig. 1E). We analyzed the motifs of lysine crotonylation through Motif analysis, and there was no obvious amino acid enrichment in Top5 (Fig. 1F).

### Differentially quantified proteins to subcellular, KOG, and functional category characterization

**Fig. 2:**
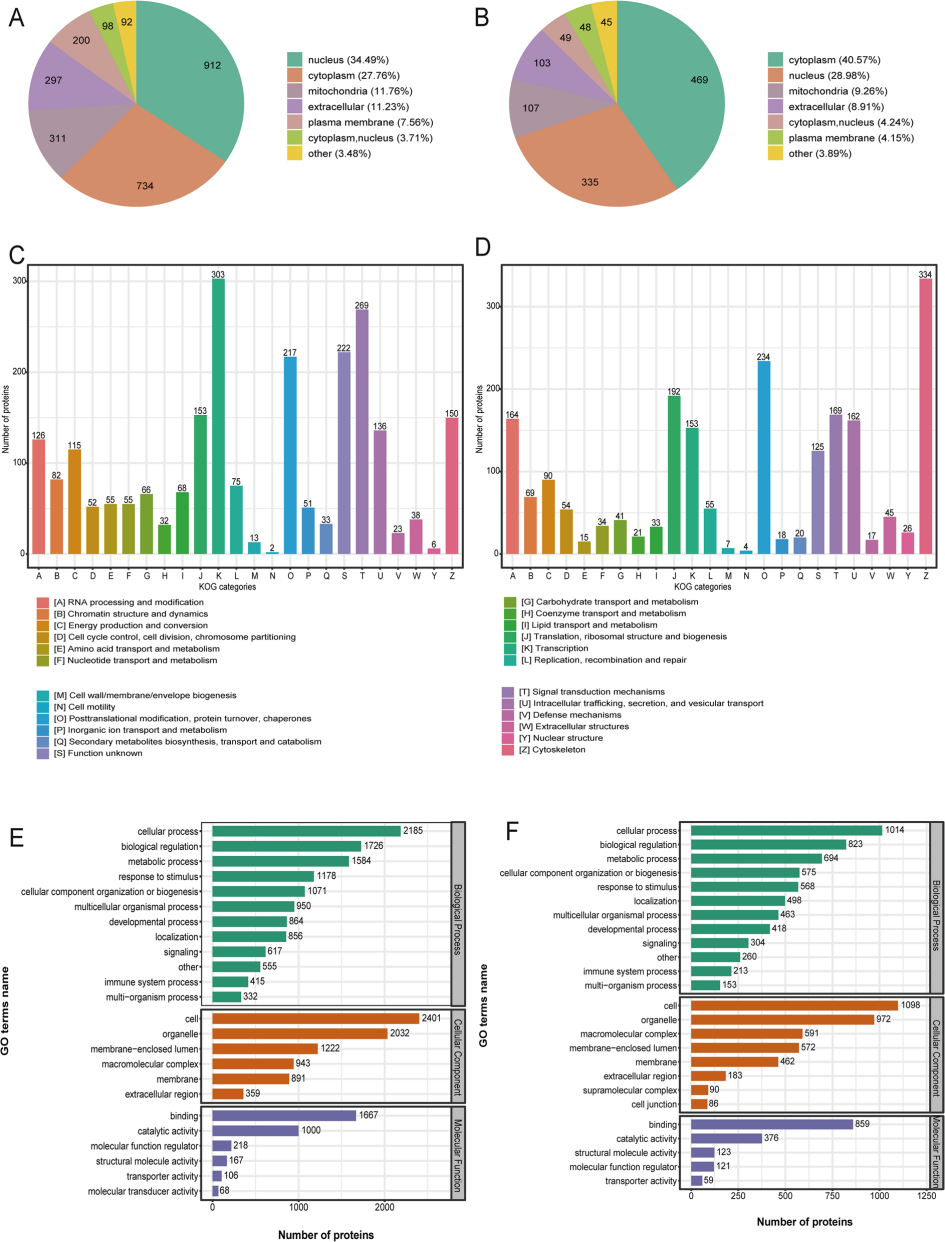
Subcellular, KOG, and Functional Category Characterization of Differentially Quantified Proteins. **A**, **B** Subcellular location of DEPs and DCPs, respectively. **C**, **D** KOG categories of DEPs and DCPs, respectively. **E**, **F** Functional category DEPs and DCPs in GO terms.

To clarify the functions of these DEPs and DCPs, we analyzed the DEPs and DCPs using subcellular location (Fig. 2A, B). We found the top three: most DEPs are nucleus (*n* = 912, 34.49%), cytoplasm (*n* = 734, 27.76%), and mitochondria (*n* = 311, 11.76%); most DCPs are cytoplasm (*n* = 469, 40.57%), nucleus (*n* = 335, 28.98%), and mitochondria (*n* = 107, 9.26%). We also investigated the KOG categories of the DEPs and DCPs (Fig. 2C, D). We found the top three: most DEPs are [K] Transcription (*n* = 303), [T] Signal transduction mechanisms (*n* = 269), and [O] Posttranslational modification, protein turnover, chaperones (*n* = 217); most DCPs are [Z] Cytoskeleton (*n* = 334), [O] Posttranslational modification, protein turnover, chaperones (*n* = 234), and [J] Translation, ribosomal structure and biogenesis (*n* = 192).

We adopted GO analysis tool to analyze DEPs and DCPs for functional category distribution (Fig. 2E, F). Three categories of GO of DEPs displayed: cellular component (92%), molecular function (81%), biological process (91%), and 24 biological groups; DCPs: functions were established for the DEPs and further divided into three classifications: cellular component (96%), molecular function (87%), biological process (94%), and 25 biological groups. Among them, most biological process of DEPs and DCPs: the cellular process (2,185 and 1,014, respectively), the biological regulation (1,726 and 823, respectively), and the metabolic process (1584 and 694, respectively). At the same, most cellular component of DEPs and DCPs: the cell (2,401 and 1,098, respectively) and the organelle (2,032 and 972, respectively). Most molecular function of DEPs and DCPs: the binding (1,667 and859, respectively) and the catalytic activity (1000 and 376, respectively).

### Functional enrichment analysis of differentially quantified proteins

**Fig. 3:**
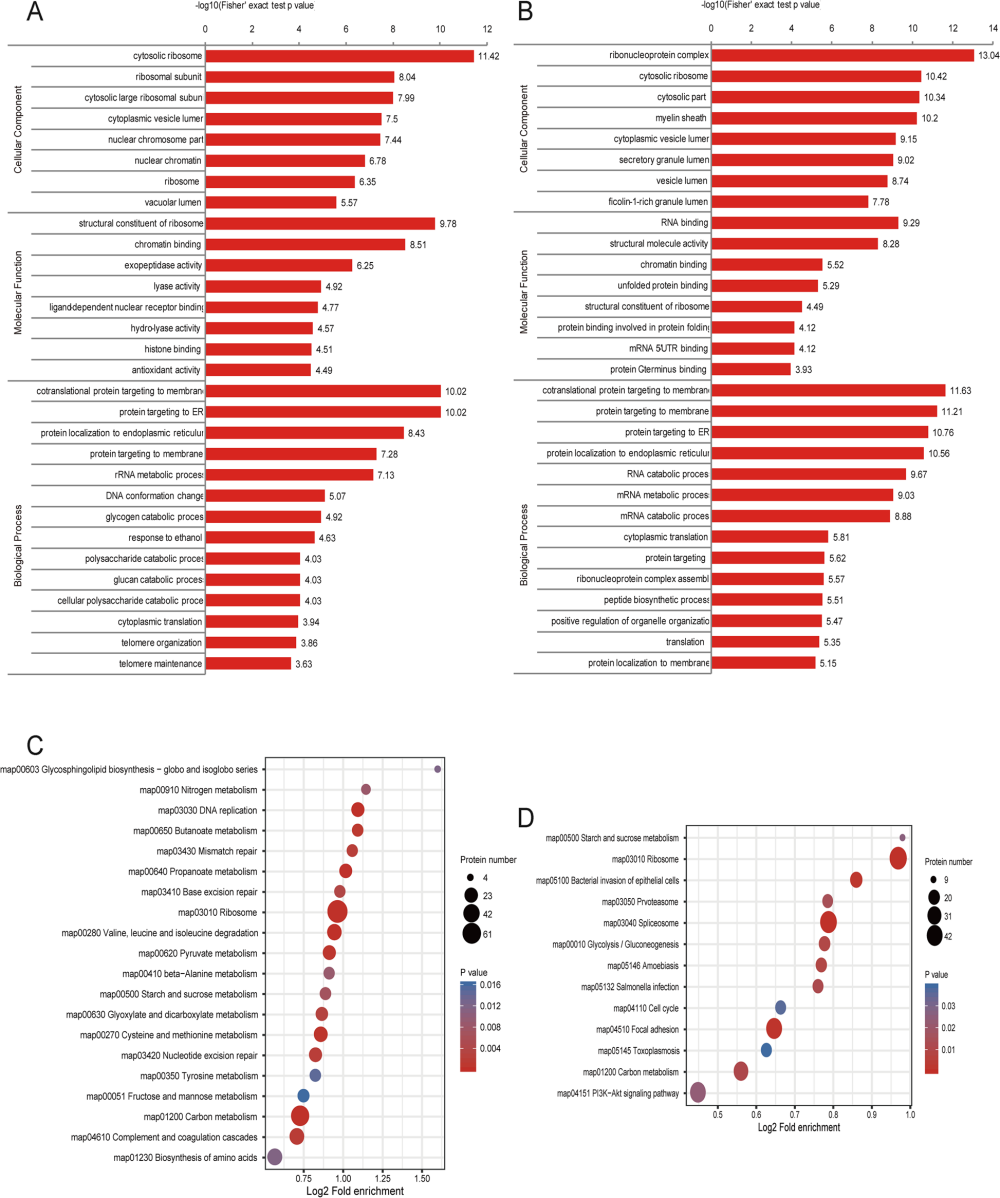
Functional Enrichment Analysis of Differentially Quantified Proteins. **A**, **B** GO enrichment analysis of DEPs and DCPs, respectively. **C**, **D** Kyoto Encyclopedia of Genes and Genomes (KEGG) functional enrichment analysis of DEPs and DCPs, respectively

For the pathways analysis of GO in DEPs, we had been enriched: cellular component (*n* = 8), molecular function (*n* = 8), and biological process (*n *= 14) (Fig. 3A). The enrichment of biological process shows that protein targeting has regulated significantly, molecular function shows that histone binding has regulated significantly, and the cellular components show significant regulation in ribosomes. For the pathways analysis of GO in DCPs, we had been enriched: cellular component (*n* = 8), molecular function (*n* = 8), and biological process (*n* = 14) (Fig. 3B). The enrichment of the biological process shows that protein targeting has regulated significantly, the molecular function shows that the chromatin binding has regulated significantly, and the cellular components show that the vesicle has regulated significantly.

In KEGG pathway of DEPs, the significant ones were in Carbon metabolism, Starch and sucrose metabolism, Nitrogen metabolism, Tyrosine metabolism, DNA replication, and so on (Fig. 3C). Regarding crotonylation, the significantones were in Carbon metabolism, Starch and sucrose metabolism, Cell cycle, Spliceosome, and so on (Fig. 3D). Furthermore, we found three same pathways (Carbon metabolism, Starch and sucrose metabolism, and Ribosome) In DEPs and DCPs.

### Metabolism regulation in developing fetal lung

**Fig. 4:**
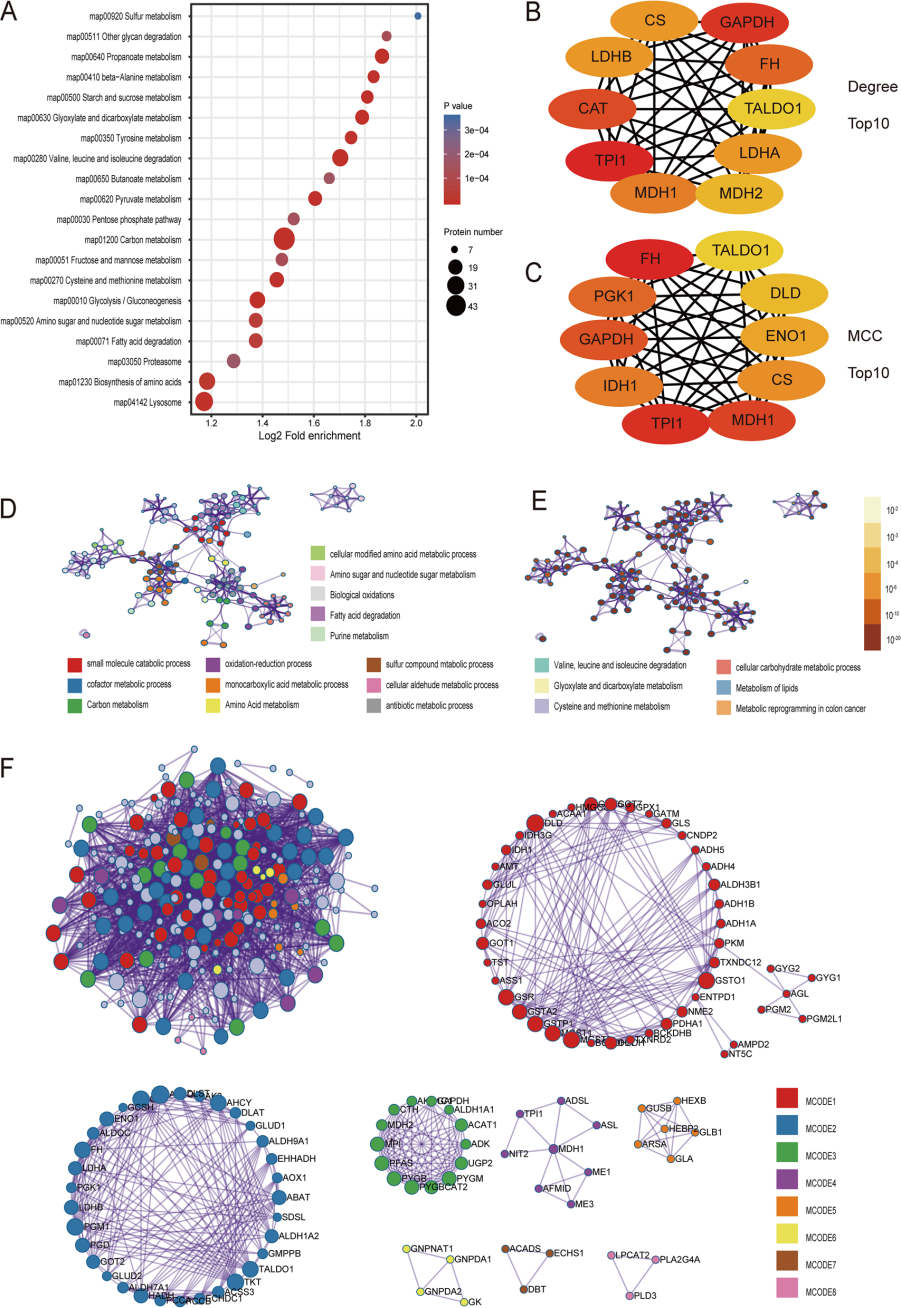
Metabolism regulation in Developing Fetal Lung. **A** KEGG functional enrichment analysis of down-regulated DEPs. **B** The Top 10 of Degree in PPI of metabolism of DEPs. (C) The Top 10 of MCC in PPI of metabolism of DEPs. **D** Metabolism of DEPs of Enrichment_GO_Color by cluster. **E** Metabolism of DEPs of Enrichment_GO_Color by *P*Value. **F** Metabolism of DEPs of PPI and MCODE by cluster

As an important part of the living organism, metabolism is especially vital in fetal development. Cell adhesion, extracellular matrix tissue, vasculature development, and lipid metabolism are the main biological processes induced in early developmental stages. Defense/immune response, ion transport, and signal pathway had identified as the main biological processes induced in the late pregnancy [5]. We found that many metabolic pathways significantly down-regulate at the protein expression level (Fig. 4A). Such as carbon metabolism, glycolysis, TCA cycle, amino acid metabolism, and lipid metabolism, and so on. The level of crotonylation modification showed that it was significantly up-regulate in carbon metabolism and glycolysis (Figure S1A). Furthermore, we analyzed the relationship between proteins regulated in metabolism and MCODE through PPI (Fig. 4D, E, F) (Table 1). Hubba analysis can look the key protein. And by selected Degree and MCC Top10 through Hubba in Cytoscape (Fig. 4B, C), we found a total of 14 core proteins, of which 12 proteins have undergone regulation of crotonylation modification site (Table 2). These results indicate that with the development of the lung, the metabolism gradually increases, and the crotonylation modification may regulate the metabolism of the fetal lung.

**Table 1:**
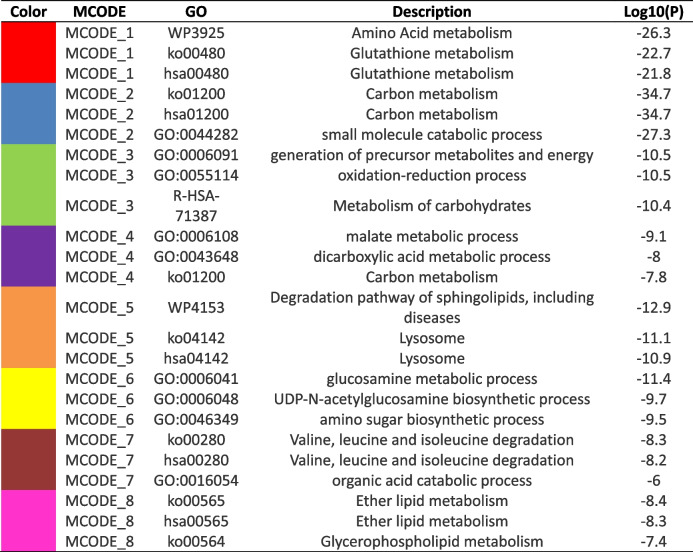
Metabolism of DEPs of PPI and MCODE by cluster

**Table 2:** Metabolism of DEPs of 12 proteins have undergone regulation of crotonylation modification site

Protein accession	Protein description	Gene name	Protein Ratio	Regulated Type	Up Sites	Down Sites
P04406	Glyceraldehyde-3-phosphate dehydrogenase	GAPDH	0.568	Down	254	334
P00338	L-lactate dehydrogenase A chain	LDHA	0.543	Down	278, 81	/
P09622	Dihydrolipoyl dehydrogenase, mitochondrial	DLD	0.536	Down	/	/
P60174	Triosephosphate isomerase	TPI1	0.46	Down	212, 96	/
P07195	L-lactate dehydrogenase B chain	LDHB	0.457	Down	233	/
P06733	Alpha-enolase	ENO1	0.442	Down	60,193,256, 406	89, 92, 103
P00558	Phosphoglycerate kinase 1	PGK1	0.429	Down	267	/
P40925	Malate dehydrogenase, cytoplasmic	MDH1	0.418	Down	/	205
P40926	Malate dehydrogenase, mitochondrial	MDH2	0.409	Down	/	/
O75390	Citrate synthase, mitochondrial	CS	0.407	Down	/	459
O75874	Isocitrate dehydrogenase [NADP] cytoplasmic	IDH1	0.388	Down	/	236
P37837	Transaldolase	TALDO1	0.382	Down	/	136, 286
P07954	Fumarate hydratase, mitochondrial	FH	0.34	Down	80	/
P04040	Catalase	CAT	0.299	Down	16	/

### Gene expression regulation in developing fetal lung

**Fig. 5:**
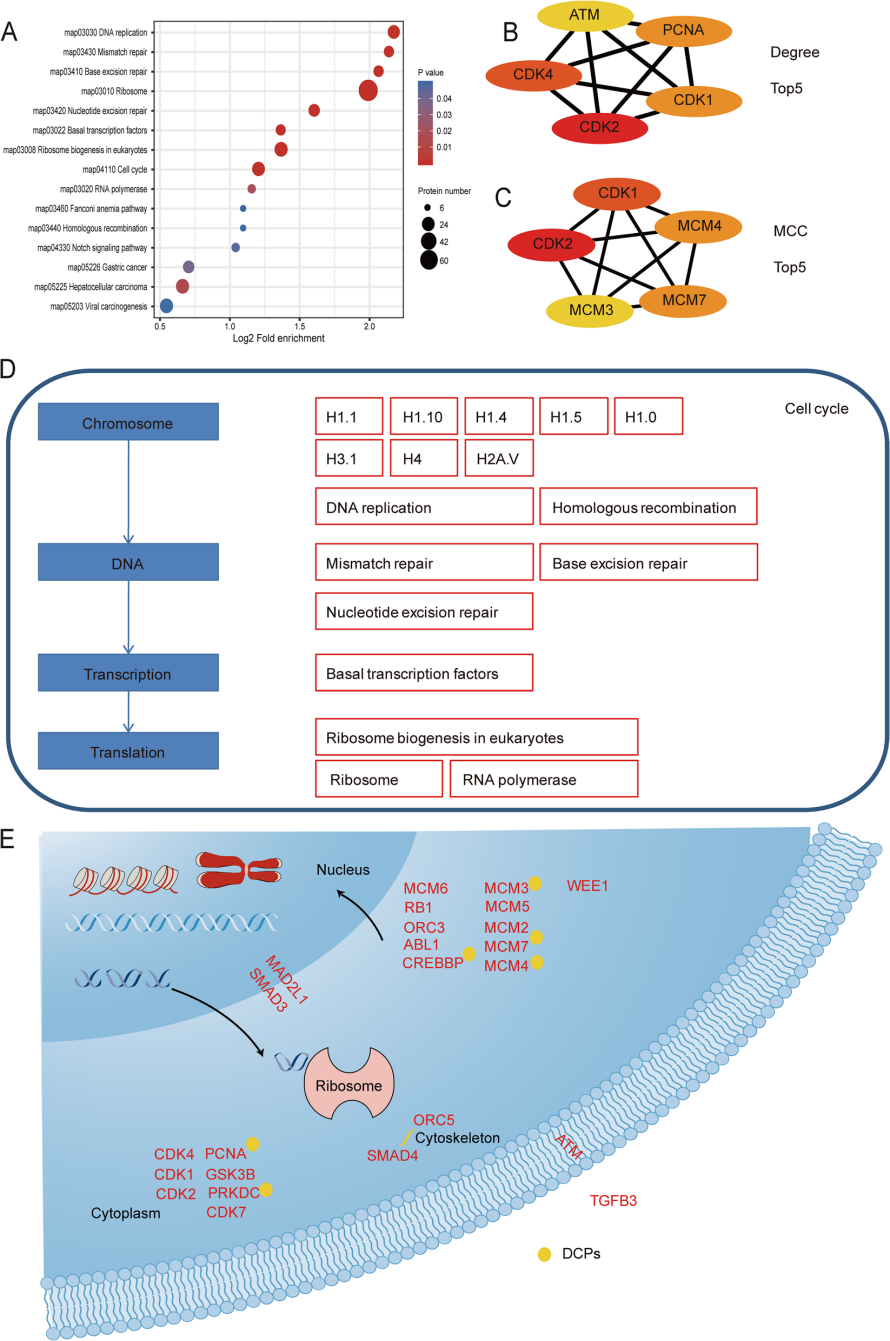
Gene Expression regulation in Developing Fetal Lung. **A** KEGG functional enrichment analysis of up-regulated DEPs. **B** The Top 5 of Degree in PPI of Cell cycle of DEPs. **C** The Top 5 of MCC in PPI of Cell cycle of DEPs. **D** Schematic diagram of gene expression of DEPs involved. **E** Schematic diagram of Cell cycle of DEPs and DCPs involved

Cell proliferation requires storage materials, and gene expression is an important part of cell proliferation and plays a decisive role in development. It had been reported that mRNA related to cell cycle, DNA repair/replication, RNA processing, and translation is the main biological process that decreases as the lung matures [5]. Our data show that Cell cycle, DNA replication, Homologous recombination, Mismatch repair, Base excision repair, Nucleotide excision repair, Basal transcription factors, RNA polymerase, Ribosome biogenesis in eukaryotes, and Ribosome are all significantly up-regulated (Fig. 5A). Meanwhile the level of crotonylation modification shows that the Cell cycle and Ribosome have enriched in DCPs (Figure S1B). Some protein sites was up-regulated, and some protein sites are down-regulated. The biological significance of crotonylation regulation needs to explore. Meanwhile we also found that the protein levels of 8 histones were up-regulated, and the crotonylation sites of 5 histones regulated to varying degrees. K55, K59, and K82 of H1.0 are down. K120 of H1.1 is up. K49, K78, and K100 of H1.5 is down, K55 is down. K47 of H1.10 is up, K75 reversly. K92 and K78 of H4 are down, K80 reversely (Fig. 5d). We further analyzed the proteins in the Cell cycle, and selected Degree and MCC Top5 through Hubba (Fig. 5B, C), and found a total of 8 core proteins, of which Cdk family proteins and Mcm proteins are the most. We found that there are more proteins of crotonylation in nuclear through subcellular localization, especially proteins related to chromatin, such as Crebbp and Mcm family proteins (Fig. 5E). However, crotonylation modification related to chromosome recombination and gene expression. Therefore, we speculate that protein crotonylation modification may affect lung development through cell proliferation.

### Lung development regulation in developing fetal lung

**Fig. 6:**
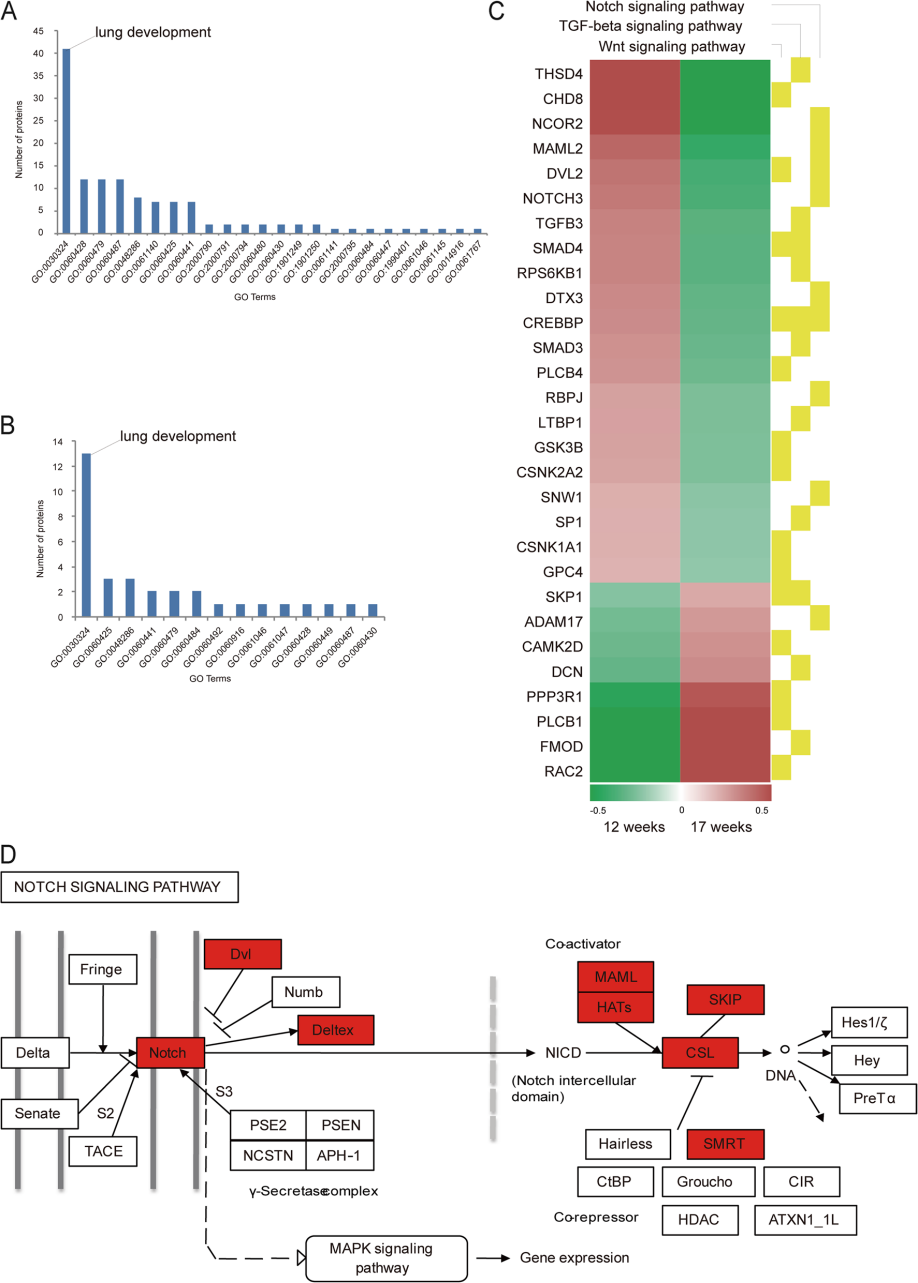
Lung Development regulation in Developing Fetal Lung. **A**, **B** GO enrichment analysis of lung related DEPs and DCPs, respectively. **C** KEGG functional enrichment analysis of (Notch, TGF-beta, and Wnt) signaling pathway DEPs and DCPs. **D** Schematic diagram of Notch signaling pathway and proteins up-regulated (red)

As the fetus develops and grows, the lungs continue to develop and improve. We found 42 DEPs and 13 DCPs protein of GO Terms regulation in various parts of the lung through protein annotation (Tables 3 and 4). Lung epithelium development, lung cell differentiation, lung epithelial cell differentiation, lung alveolus development, lung secretory cell differentiation, lung morphogenesis, and epithelial tube branching involved in lung morphogenesis are more proteins regulation. There are also modifications in these biological processes. We found 24 and 14 terms of biological process related to lungs in DEPs and DCPs (Fig. 6A, B). Among them, the 41 DEPs and 13 DCPs are in the term of lung development. As a result, the four proteins (Hmgb1, Cux1, Itga3, and Asah1) have dysregulated in expression and crotonylated of protein. For example, in the nucleus, Hmgb1 is one of the major chromatin-associated non-histone proteins. It acts as a DNA chaperone involved in replication, transcription, chromatin remodeling, V(D)J recombination, DNA repair, and genome stability [10]. At the same time, we also found the regulation in transcription factor–for instance, Foxp1, Foxp4, Sox9, Nfib, Gata6, and Nkx2-1. Nfib, Gata6, and Nkx2-1 are participating in many lungs of GO terms. Regarding protein of crotonylation of the lung, Ctnnb1 and Yap1 of crotonylation protein are participating in many GO terms of lungs.

**Table 3:** Lung development regulation of 42 DEPs in developing fetal lung

Protein accession	Protein description	Gene name	Ratio	Regulated Type
P25391	Laminin subunit alpha-1	LAMA1	11.154	Up
P48436	Transcription factor SOX-9	SOX9	5.506	Up
Q8IVH2	Forkhead box protein P4	FOXP4	3.686	Up
Q92908	Transcription factor GATA-6	GATA6	3.591	Up
Q63HK5	Teashirt homolog 3	TSHZ3	3.468	Up
P20336	Ras-related protein Rab-3A	RAB3A	3.028	Up
Q9Y618	Nuclear receptor corepressor 2	NCOR2	2.975	Up
P20719	Homeobox protein Hox-A5	HOXA5	2.412	Up
Q02447	Transcription factor Sp3	SP3	2.286	Up
P09429	High mobility group protein B1	HMGB1	2.279	Up
P10600	Transforming growth factor beta-3 proprotein	TGFB3	2.008	Up
Q6NYC1	Bifunctional arginine demethylase and lysyl-hydroxylase JMJD6	JMJD6	1.94	Up
O00712	Nuclear factor 1 B-type	NFIB	1.845	Up
P84022	Mothers against decapentaplegic homolog 3	SMAD3	1.838	Up
P51608	Methyl-CpG-binding protein 2	MECP2	1.793	Up
Q9H334	Forkhead box protein P1	FOXP1	1.763	Up
Q99593	T-box transcription factor TBX5	TBX5	1.74	Up
P57082	T-box transcription factor TBX4	TBX4	1.72	Up
Q06330	Recombining binding protein suppressor of hairless	RBPJ	1.669	Up
P04150	Glucocorticoid receptor	NR3C1	1.661	Up
O94880	PHD finger protein 14	PHF14	1.644	Up
Q13948	Protein CASP	CUX1	1.624	Up
P43699	Homeobox protein Nkx-2.1	NKX2-1	1.591	Up
P39880	Homeobox protein cut-like 1	CUX1	1.581	Up
Q9Y547	Intraflagellar transport protein 25 homolog	HSPB11	1.533	Up
P08047	Transcription factor Sp1	SP1	1.528	Up
Q9UPY3	Endoribonuclease Dicer	DICER1	1.527	Up
P09486	SPARC	SPARC	0.661	Down
Q16706	Alpha-mannosidase 2	MAN2A1	0.621	Down
P29466	Caspase-1	CASP1	0.613	Down
P24821	Tenascin	TNC	0.575	Down
Q14118	Dystroglycan	DAG1	0.528	Down
O95994	Anterior gradient protein 2 homolog	AGR2	0.395	Down
P63098	Calcineurin subunit B type 1	PPP3R1	0.362	Down
P38571	Lysosomal acid lipase/cholesteryl ester hydrolase	LIPA	0.316	Down
P09960	Leukotriene A-4 hydrolase	LTA4H	0.277	Down
P54868	Hydroxymethylglutaryl-CoA synthase, mitochondrial	HMGCS2	0.27	Down
P26006	Integrin alpha-3	ITGA3	0.251	Down
O94788	Retinal dehydrogenase 2	ALDH1A2	0.25	Down
Q9UBR2	Cathepsin Z	CTSZ	0.248	Down
Q13510	Acid ceramidase	ASAH1	0.216	Down
P12821	Angiotensin-converting enzyme	ACE	0.199	Down

**Table 4:** Lung development regulation of 13 DCPs in developing fetal lung

Protein accession	Gene name	Protein description	Position	Amino acid	Ratio	Regulated Type
O15230	LAMA5	Laminin subunit alpha-5	2481	K	0.598	Down
P09429	HMGB1	High mobility group protein B1	141		0.365	Down
			59		0.25	Down
P22626	HNRNPA2B1	Heterogeneous nuclear ribonucleoproteins A2/B1	3		0.194	Down
P26006	ITGA3	Integrin alpha-3	471		2.295	Up
P28482	MAPK1	Mitogen-activated protein kinase 1	340		2.399	Up
P35222	CTNNB1	Catenin beta-1	671		2.182	Up
P39880	CUX1	Homeobox protein cut-like 1	189		2.08	Up
			117		0.544	Down
P46937	YAP1	Transcriptional coactivator YAP1	315		2.2	Up
Q13308	PTK7	Inactive tyrosine-protein kinase 7	852		4.891	Up
			788		2.918	Up
Q13510	ASAH1	Acid ceramidase	310		0.502	Down
Q15185	PTGES3	Prostaglandin E synthase 3	65		1.956	Up
			35		1.75	Up
Q68CZ2	TNS3	Tensin-3	1170		2.03	Up
Q9UP38	FZD1	Frizzled-1	273		1.643	Up

Six signaling pathways are involved in lung organogenesis, such as Notch, Tgfβ / Bmp, Sonic hedgehog (Shh), Fgf, Egf, and Wnt [11]. Studies have reported that the predicted inhibitory role of Notch signaling during lung maturation [5]. Our data is also significantly enriched in the Notch signaling pathway and up-regulate (Fig. 6D). In KEGG analysis, the 14 DEPs and 9 DCPs belong to the Wnt signaling pathway (Fig. 6C). Crebbp is only both dysregulated. The protein is up to expression and the position of K1524 occurred down in modification. Meanwhile, we found the 9 DEPs and 2 DCPs belong to Notch signaling pathway. Wherein, Crebbp is only both dysregulated. We also found the 11 DEPs and 7 DCPs belong to the Tgf-beta signaling pathway. The Crebbp and Dcn are both dysregulated. Therefore, Crebbp is the role of importance in these pathways; it maybe regulates lung development. In the PPI network of all the dysregulated proteins of expression, Crebbp interacts with many proteins. Maybe it can influence all the dysregulated proteins of expression. Hence, we suggest that Crebbp is a key protein of lung organogenesis; it can be a biomarker to detect lung development.

## Discussion

In the present study, we performed LC–MS/MS-based proteomic analysis. We identified 2,645 DEPs and 1,156 DCPs between the fetal lung of 17 weeks and 12 weeks and 514 proteins with both expression and crotonylation difference. The lungs of the fetus at 12 weeks are in Pseudoglandular and at 17 weeks are in Canalicular. We revealed the regulation of protein related to lung development from the differences between the two phases.

Energy metabolism not only guarantees the metabolism of the fetus, but it is also the basis for the growth and development of fetal tissues and organs. Several new protein post-translational modifications that use intermediates in metabolism have been discovered, and interestingly, several of these modifications, in turn, regulate the activity of metabolic enzymes [8]. We analyzed the metabolic regulation of different proteins, in which lipid metabolism is consistent with what has been reported [5]. Our data shows that multiple metabolic pathways enhanced with the development of the lung. Secondly, we also found 11 core proteins from various metabolically regulated proteins, and most of these core proteins have undergone crotonylation modification [9]. Here, we suggest that if you need to study the metabolism of the lungs, you can start from these aspects, and you can carry out in-depth research from the perspective and direction of crotonylation modification.

Cell proliferation and differentiation are the conditions for the growth of fetal tissues and organs, and the basis for their functional development [12]. In the process of cell proliferation, the most involved is the gene expression in the cell. The results revealed by our data are consistent with previous reported. As the lungs develop, cell proliferation related protein express down, and we speculate it maybe decrease the rate of cell proliferation. Among them, Cdk2 and Cdk1 had reported as important genes in cell cycle control, and they are the core proteins in our cell cycle as same [4]. Secondly, our mini-chromosome maintenance protein Mcm family is at the core position [5]. It is reported that it decreases with development, and most of the proteins show regulation in crotonylation modification. We speculate that the crotonylation modification may affect the epigenetics of lung development through the Mcm family. The crotonylation firstly be found on histones and was related to genetic regulation [13]. Our data also detected regulation in the level of crotonylation modification in H1 and H4. These proteins may regulate some epigenetic change–specific needs further research.

Lung development has regulated by transcription factor [12]. Transcription factors determine the correct fate and differentiation of lung epithelial cells. We analyzed transcription factors related to lung development. Nkx2-1 is located at the center of the transcription network, which is essential for imposing the fate of lung epithelial cells through activation and inhibition mechanisms. Our data showed that Nkx2-1 expressed at 12 and 17 weeks, and it down regulated in lung development. A member of the Gata family of zinc finger proteins, highly expressed in the lung’s endoderm and vascular smooth muscle, and the loss of lung epithelial-specific Gata6 during development leads to defective epithelial differentiation and increased proliferation [14]. Our data show that Gata6 expression is present at 12 weeks and 17 weeks, and it down regulated with the development of lungs like Nkx2-1 [15]. We speculate that they have the same interaction relationship as reported in the literature. The Fox protein family plays an important role in many aspects of lung development [16]. Foxp1, Foxp2, and Foxp4 expressed in overlapping patterns in the developing lung and the postnatal lung. Our data show that Foxp1 and Foxp4 down regulated with lung development. There are also reports that Sox9 plays multiple roles in lung epithelial cells during branch morphogenesis, and Sox9 is essential for correct branch morphogenesis [17]. Our data show that Sox9 expression is at 12 and 17 weeks, and it down regulated with lung development. Hmgb protein is a non-histone chromatin-related molecule that affects transcription and cell differentiation through histone binding and RNA polymerase II activity [18]. Our data show that Hmgb1 has undergone crotonylation modification. Combined with the role of this protein, we believe that can study the epigenetic mechanism of lung development through Hmgb1.

Non-neurologically regulated body fluids or secreted factors, including growth factors or hormones, are one of the main factors that regulate the growth process of the fetus in the uterus. The molecular signaling pathways that regulate the different stages of lung development studied. Tgf-β, Wnt, hedgehog, Notch, and fibroblast growth factor (Fgf) signaling pathways are involved in regulating lung size, branching, and pattern during lung development. Our KEGG analysis enriched the Tgf-β, Wnt, and Notch signaling pathways. The expression of the three Tgf-β gene isoforms, receptors (I, II, and III), and signal transduction mediators (Smad-2, -3, -4, -6, and -7) are present throughout lung development [19]. Our data show that Smad3 and Smad4 in this signaling pathway different expressed as the lungs develop. In mice, Tgf-β1 showed to inhibit branch morphogenesis. Up-regulation of Tgf-β receptor II or Smad-2, -3, -4, and -6 also showed the effect of inhibiting branch morphogenesis. Our data showed that Smad3 and Smad4 had down-regulate with lung development, which can promote branch morphology Formation to meet the normal developmental needs of the lungs. The expected inhibitory effect is Notch signaling during lung maturation. Our data also shows that most of the proteins in the Notch signaling pathway are down-regulate with development. Crebbp gene encodes Creb-binding protein, Acetylates histones, giving a specific tag for transcriptional activation [20]. It also acetylates non-histone proteins, like Ddx21, Fbl, Irf2, Mafg, Ncoa3, Polr1e, Paf53, and Foxo1 [21], and Functions as a transcriptional coactivator for Smad4 in the Tgf-beta signaling pathway [22]. Because of a common protein has three important signal pathways, it also exists in the cell cycle and linked to various proteins in PPI. We suggest that it may be used to an important protein for lung research in the future.

However, there were some limitations to consider in the current study. First, it is a small number size cross-sectional study. Second, the results of DEPs and DCPs were two different samples at different developmental periods. The LC–MS/MS measurement could have neglected individual information. Third, although thousands of proteins had detected, there were no more than one thousand proteins with expression and crotonylation quantitative data. Some key proteins could have neglected key information. The most studied Fgf signaling pathway and Fgf10 expression of the protein were not detected in the lung [23]. Future studies should explore these limiting factors in more detail.

## Conclusion

The quantitative proteomic analysis revealed differentially expressed and crotonylated proteins between 12 and 17 weeks of the fetal lung. The key features of DEPs and DCPs indicate that lung development is a complex process. We were the first to report on the epigenetic characteristics of fetal lung development and found some key proteins from it, laying a certain foundation for follow-up related research. Simultaneously, it can also be used as a reference for some clinical development-related diagnoses or provide candidate markers for diagnosis and treatment targets for the treatment of pulmonary dysplasia.

### Details of material usage and methods

#### Sample collection and processing

All the schemes in this study are in line with the Interim Measures for the Management of Human Genetic Resources of the Ministry of Science and Technology of China. The ethics committee approved the human embryo collection and research protocol of Shenzhen People's Hospital (LL-KY-2019591). This study strictly abides by Shenzhen People’s Hospital’s laws and institutional ethics regulations on selective termination of pregnancy specimens. After the donor patients signed the informed consent document, the lung tissue samples of the developing fetus had collected. Process the tissue sample immediately after separation. Transfer the tissue to a sterile 10 cm2 tissue culture dish, cut into pieces < 0.6 cm3, and then transfer to a 50 mL conical tube.

#### Protein extraction

Under the guidance of experimental method by Xu et al [24], we carried out protein extraction. In short, prepare liquid nitrogen, lysis buffer A (8 M urea, 1% Protease Inhibitor Cocktail) for Proteomics, lysis buffer B (8 M urea, 1% Protease Inhibitor Cocktail, 3 μM TSA, 50 mM NAM) for Crotonylation and a mortar (pre-cooled with liquid nitrogen in advance) and an ultrasound system. Remove the sample from -80 °C, weigh 5 mg of tissue sample into a mortar, and then add liquid nitrogen to grind until the sample becomes a powder. Next, divide the sample into two parts 1:4, add the smaller part with 4 times the powder lysis buffer A, and the larger part with 4 times the powder lysis buffer B, and lyse the tissue by ultrasound. Finally, the cell debris was removed (4 °C, 12,000 g centrifugation for 10 min), and the protein concentration of the supernatant was determined using the BCA kit.

BCA Reagent A, 500 ml, containing sodium carbonate, sodium bicarbonate, bicinchoninic acid and sodium tartrate in 0.1 M sodium hydroxide.

BCA Reagent B, 25 ml, containing 4% cupric sulfate Albumin Standard Ampules, 2 mg/ml, 10 × 1 ml ampules, containing bovine serum albumin (BSA) at 2.0 mg/ml in 0.9% saline and 0.05% sodium azide.

#### Trypsin digestion

We used our own experimental method to trypsin digestion by Lu et al [25]. Overall, prepare 5 mM dithiothreitol and 11 mM iodoacetamide for alkylation, 100 mM triethylammonium bicarbonate (TEAB) buffer (Sigma-Aldrich; Merck KGaA). First, the protein solution was reduced with 5 mM dithiothreitol for 30 min at 56 °C, and then diluted with TEAB to obtain a urea concentration of < 2 M. Finally, digest twice (the first digestion is overnight at 37 °C with a trypsin to protein mass ratio of 1:50, and the second digestion is 4 h at 1:100).

#### Affinity enrichment

We adopted our own experimental method to affinity enrichment by Lin et al [26]. Prepare NETN buffer (100 mm NaCl; 1 mm EDTA; 50 mm Tris–HCl; 0.5% NP-40; pH 8.0) to enrich the crotonylated peptides. Dissolve trypsin peptides and pre-washed antibody beads (PTM Biolabs, Inc.) in NETN buffer, incubate gently at 4 °C, and shake overnight. Wash the beads four times (with NETN buffer) and then twice (with H2O). Next is the classification, drying and desalting of the peptides. The peptides bound to the beads were eluted (using 0.1% trifluoroacetic acid), combined and vacuum dried, and the resulting peptides were desalted (using C18 ZipTips (EMD Millipore) for liquid chromatography-MS (LC–MS/MS) analysis).

#### LC–MS/MS analysis

We use the EASY-nLC 1000 ultra-high performance liquid chromatography system (Thermo Fisher Scientific, Inc.) and the Q Exactive Plus instrument of the ultra-high performance liquid chromatograph (Thermo Fisher Scientific, Inc.). To prepare solvent A (0.1% formic acid reagent), Solvent B (0.1% formic acid in 98% acetonitrile) and a self-made reversed-phase analytical column (length, 15 cm; ID 75 µm), used to dissolve tryptic peptides, and load the sample onto the reversed-phase analytical column. Settings: chromatographic system (at a constant flow rate of 400 nL/min), gradient: solvent B, increased from 6 to 23% in 26 min, increased from 23 to 35% in 8 min, increased to 80% in 3 min and then maintained at 80% in the last 3 min. Use nanospray positive ion source to process peptides, MS/MS analysis: connect Q Exactive Plus instrument online; and settings: 2.0 kV (electrospray voltage) 350–1 (m/z scan range). The first step is to check 800 complete scans and complete peptides (using Orbitrap with a resolution of 70,000), and the second step is to set 28 (standard collision energy) MS/MS peptides, and analyze fragments (using Orbitrap with a resolution of 17,500). Rate), the third step is to perform the data-related process (alternating between MS scan and 20 MS/MS scans and 15.0 s of dynamic exclusion), and finally, set the automatic gain control to 5E4 and the fixed first quality to 100 m/z.

#### Database search

Trypsin/P is designated as a lyase and allows ≤ 4 deletions to be cleavage. Search engine: Maxquant search engine (v.1.5.2.8), crotonylation database (https://www.uniprot.org/), analyze MS/MS data, and connect with reverse decoy database to search serial MS data. Search settings: the first time 20 ppm (mass tolerance of precursor ions), 5 ppm (main search), 0.02 Da (mass tolerance of fragment ions). It based on fixed modification (carbamylmethylation of cystine) and variable modification (crotonylation and oxidation of methionine). For the false discovery rate, we adjusted it to < 1% and set the minimum score to > 40.

#### Bioinformatics analysis to proteins

We refer to the analysis method of Chen et al [27]. First, perform gene ontology (GO) annotation by UniProt-GOA database (www. http://www.ebi.ac.uk/GOA/, gaf-version, 2.0) on all identified proteins, subcellular localization by WoLF PSORT (https://www.genscript.com/wolf-psort.html), and annotation of kyoto encyclopedia of genes and genomes (KEGG) pathway. Then, we performed GO and KEGG functional enrichment of the protein (statistical method: Two-tailed Fisher's exact test). Finally, we use the one-way hierarchical clustering in Genesis to visualize the cluster members through the heatmap using the "heatmap.2" function in the "gplots" R-package.

#### Motif analysis

We adopted the motif analysis method of Huang et al [28]. We used Motif-x software (http://motif-x.med.harvard.edu/) to model 21-mers (10 amino acids upstream and downstream of the site), and all identified crotonylated modified peptides Sequence analysis. All protein sequences in the database used to background database parameters, and the default values used for other parameters.

#### Protein–protein interaction analysis

Protein–protein interaction (PPI) had conducted using a procedure described by Huang et al [28]. Identified proteins had performed by PPI analysis using Cytoscape software (Version 3.3.0). The search tool for retrieving mutual genes/proteins (STRING) database (the confidence score by using a metric) to define interaction confidence can obtain PPI networks. We fetched high confidence (confidence score ≥ 0.9) of all mutual. Among them, MCC and Degree are good tools to analyze core proteins.

### Supplementary Information

**Additional file 1: Figure S1.** (A) KEGG functional enrichment analysis of up-regulated DCPs. (B) KEGG functional enrichment analysis of DCPs.

## Data Availability

Uploading.
