# Supplementary figures and images for: Systematic proteomics profiling of lysine crotonylation of the lung at Pseudoglandular and Canalicular phases in human fetus
Source: Proteome Sci. 2023 Dec 1;21:22. doi: 10.1186/s12953-023-00215-8 (PMC10691156; doi:10.1186/s12953-023-00215-8)

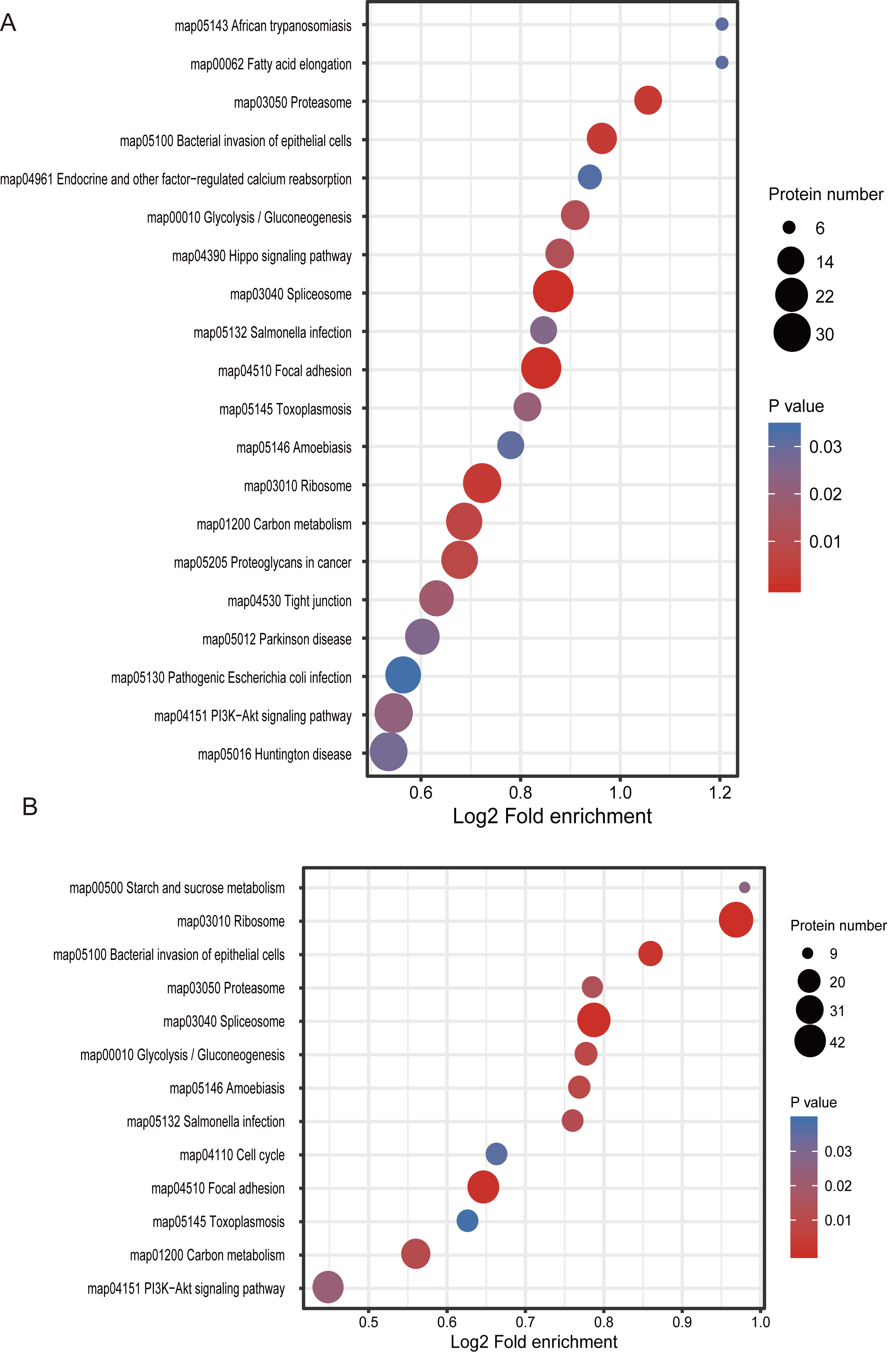

Supplement: Supplementary file 1 — Additional file 1: Figure S1. (A) KEGG functional enrichment analysis of up-regulated DCPs. (B) KEGG functional enrichment analysis of DCPs. [file 12953_2023_215_MOESM1_ESM.tif]
